# Targeting enolase 1 reverses bortezomib resistance in multiple myeloma through YWHAZ/Parkin axis

**DOI:** 10.1186/s12929-024-01101-x

**Published:** 2025-01-20

**Authors:** Xuejie Gao, Qilin Feng, Qikai Zhang, Yifei Zhang, Chaolu Hu, Li Zhang, Hui Zhang, Guanli Wang, Ke Hu, Mengmeng Ma, Zhuning Wang, Yujie Liu, Dong An, Hongfei Yi, Yu Peng, Xiaosong Wu, Gege Chen, Xinyan Jia, Haiyan Cai, Jumei Shi

**Affiliations:** 1 Department of Hematology, Shanghai East Hospital, Tongji University School of Medicine, Shanghai, 200120 China; 2 Department of Hematology, Affiliated Hospital of Nantong University, Jiangsu, 226001 China; 3 State Key Laboratory of Cardiovascular Diseases and Medical Innovation Center, Shanghai East Hospital, School of Medicine, Tongji University, Shanghai, 200120 China

**Keywords:** Multiple myeloma, ENO1, Mitophagy, YWHAZ, Chemoresistance

## Abstract

**Background:**

Enolase 1 (ENO1) is a conserved glycolytic enzyme that regulates glycolysis metabolism. However, its role beyond glycolysis in the pathophysiology of multiple myeloma (MM) remains largely elusive. Herein, this study aimed to elucidate the function of ENO1 in MM, particularly its impact on mitophagy under bortezomib-induced apoptosis.

**Methods:**

The bone marrow of clinical MM patients and healthy normal donors was used to compare the expression level of ENO1. Using online databases, we conducted an analysis to examine the correlation between ENO1 expression and both clinicopathological characteristics and patient outcomes. To investigate the biological functions of ENO1 in MM and the underlying molecular mechanisms involved, we conducted the following experiment: construction of a subcutaneous graft tumor model, co-immunoprecipitation, western blot, quantitative real-time polymerase chain reaction, immunohistochemistry, flow cytometry, and cell functional assays.

**Results:**

ENO1 was identified as an unfavorable prognostic factor in MM. ENO1 knockdown suppresses tumorigenicity and causes cell cycle arrest. Inhibition of ENO1-regulated mitophagy sensitizes tumor cells to apoptosis. ENO1 enhanced the stability of the YWHAZ protein by increasing the acetylation of lysine in YWHAZ while antagonizing its ubiquitination, which in turn promoted mitophagy. HDAC6 mediates the deacetylation of YWHAZ by deacetylating the K138 site of YWHAZ. Inhibition of HDAC6 increased YWHAZ acetylation and decreased YWHAZ ubiquitination. Furthermore, combination treatment with bortezomib and pharmaceutical agents targeting ENO1 has synergistic anti-MM effects both in vivo and in vitro.

**Conclusion:**

Our data suggest that ENO1 promotes MM tumorigenesis and progression. ENO1 activates mitophagy by promoting the stability of YWHAZ and inhibits apoptosis and thus, leads to the drug resistance. ENO1-dependent mitophagy promotes MM proliferation and suppresses the level of bortezomib-induced apoptosis. Inhibition of ENO1 may represent a potential strategy to reverse the resistance of MM to bortezomib.

**Supplementary Information:**

The online version contains supplementary material available at 10.1186/s12929-024-01101-x.

## Introduction

Multiple myeloma (MM) is the second most common haematological malignancy, and often presents with asymptomatic precursor conditions [1]. It has been reported that the global incidence of MM has increased in developed countries, particularly among men aged 50 years or over [2, 3]. Despite significant progress in diagnostics and therapeutics, the prognosis for patients with MM remains grim. Patients with MM usually experience recurrence, drug resistance, and an unfavourable prognosis [4]. Hence, it is imperative to discern novel prognostic indicators and decipher their underlying molecular mechanisms to develop therapeutic targets for MM.

Enolase 1 (ENO1) is a conserved glycolytic enzyme that catalyses the conversion of 2-phosphoglycerate to phosphoenolpyruvate [5]. There is growing evidence that ENO1 is a multifunctional tumor-related protein besides its primary function in glycolysis, and is involved in a variety of physiological processes, including growth control, hypoxia tolerance and autoimmune activity [6–8]. Therefore, exploring and identifying the downstream targets and elucidating the molecular mechanism underlying chemoresistance triggered by aberrant ENO1 expression carry immense clinical significance.

Mitophagy and apoptosis are two interconnected cellular processes that play critical roles in maintaining cellular homeostasis and regulating cell fate. Mitophagy refers to the selective autophagic process that is mainly responsible for removing and degrading damaged or dysfunctional mitochondria [9]. Apoptosis, on the other hand, is a highly regulated form of programmed cell death. When mitophagy is impaired, damaged mitochondria cannot be removed, and the cell inevitably undergoes apoptosis. In addition, inhibition of apoptosis may enable cancer cells to escape chemotherapeutic drug-induced death, thereby promoting progression and chemoresistance in MM [10, 11]. A deep understanding of the relationship between mitophagy and apoptosis and its regulatory mechanism is of great significance for revealing the mysteries of cell death and developing new therapeutic strategies.

YWHAZ, also referred to as 14-3-3ζ, belongs to the 14-3-3 protein family and is a central hub protein in signal transduction pathways and tumour progression [12, 13]. It regulates mitophagy by interacting with key proteins such as Parkin [14–16]. Additionally, the interaction of YWHAZ with several apoptosis-related proteins suggests that YWHAZ plays a pivotal role in modulating apoptosis and facilitating cellular adaptability in response to environmental stresses [17–19]. Furthermore, covalent post-translational modifications (PTMs) are important cellular regulatory mechanisms by which the activity of proteins can be controlled and there is extensive crosstalk between PTMs [20]. It is now becoming apparent that cross-talk between two protein lysine modifications, acetylation and ubiquitination, serves as a crucial regulatory mechanism for essential cellular functions. One of the most notable effects is that lysine acetylation inhibits proteasome-mediated protein degradation [21–23].Crosstalk between the acetylation and ubiquitination might determine whether YWHAZ is stabilized or marked for degradation, impacting its contribution to critical cellular processes such as growth, apoptosis, and mitophagy [24].

In this study, we investigated the underlying mechanism by which the aberrant expression of ENO1 inhibits the apoptotic process by activating mitophagy. We reported for the first time that YWHAZ is a crucial downstream functional factor and therapeutic target of ENO1. ENO1 affects the protein stability of YWHAZ by regulating its PTM and activating mitophagy, thereby conferring resistance to MM chemotherapy. These findings suggest that ENO1 may be a potential therapeutic target for MM treatment.

## Materials and methods

### Clinical samples

Bone marrow samples were obtained from patients with MM after obtaining written informed consent at the Department of Hematology, Shanghai East Hospital (Shanghai, China). The protocol for the collection and usage of clinical samples was approved by the Shanghai East Hospital Ethics Committee, the approval number is “2024YS-234”.

## Cells, antibodies and reagents

The human multiple myeloma cell lines NCI-H929, RPMI-8226, and HEK293T cells were purchased from the American Type Culture Collection (Manassas, VA, USA), and were cultured in RPMI-1640 or Dulbecco’s modified Eagle’s medium supplemented with 10% fetal bovine serum (catalogue no. 16000-044; Gibco, Grand Island, NY, USA) at 37 °C in a humidified atmosphere containing 5% CO_2_. The bortezomib (BTZ)-resistant cell lines H929R and 8226R5 have been described in our previous articles [25, 26]. To maintain the drug resistance of the BTZ-resistant cell lines, H929R and 8226R5 were cultured in the presence of 20 nM BTZ. The primary antibodies including cleaved caspase-8, cleaved caspase-9, cleaved caspase-3, p62, HA-tag, CDK4, CDK6, CyclinD1, β-actin and DYKDDDDK Tag (Flag) were purchased from Cell Signaling Technology (Beverly, MA, USA); LC3B and ENO1 were purchased from Abcam (Cambridge, MA, UK); Parkin and YWHAZ were purchased from Proteintech (Wuhan, Hubei, China). The chemotherapeutic reagents, AP-III-a4, oligomycin, nicotinamide (NAM), trichostatin A (TSA), Tubastatin A HCl, and cycloheximide (CHX) were purchased from MedChemExpress (Monmouth Junction, NJ, USA). Bortezomib and Puromycin were purchased from Sigma (St. Louis, MO, USA).

## RNA interference, plasmids and transfection

All transfections were performed using Lipofectamine™ 3000 (Invitrogen, L3000015) according to the manufacturer’s instructions. The respective primers sequence for ENO1 knockdown are listed in the supplementary file: Table S2. The details are described in the supplementary materials and methods.

## Cytotoxicity assay, cell apoptosis and cell cycle assay

Cell cytotoxicity was assessed using a Cell Counting Kit-8 (CCK-8) (Dojindo, Kumamoto, Japan). Cell apoptosis was determined by staining with FITC Annexin V and propidium iodide (PI) using the FITC Annexin V Apoptosis Detection Kit I (BD Biosciences, CA, USA). For the cell cycle assay, cells were subjected to serum-free starvation followed by PI (BD Biosciences, CA, USA) staining and analyzed using flow cytometry. Data analysis was conducted using FlowJo software (TreeStar, Ashland, OR, USA). All assays were performed in triplicate.

## RNA isolation and relative quantitative PCR

RNA was isolated from cells with the RNA-Quick purification kit (ES Science, Shanghai, China) as advised by the manufacturer. 1000 ng of purified mRNA was subjected to reverse transcription (RT) by utilizing the Reverse transcription kit (TaKaRa, Shiga, Japan) according to the manufacturer’s instructions. Quantification of gene expression was performed using a 7900HT Fast real-time PCR system and associated SDS software (Applied Biosystems). The thermal profile of the reaction consisted of an initial denaturation at 95 °C for 3 min followed by 40 cycles at 95 °C for 3 s and at 60 °C for 30 s. Amplification of the sequence of interest was normalized with a housekeeping gene (ACTB). Fold-change values were calculated using the ΔΔCt method. Specific primers for ENO1, YWHAZ, DLOOP, 12 S, and ACTB are shown in Supplementary Table 1.

### TUNEL assay

Cell apoptosis was assessed by TUNEL staining according to the manufacturer’s instructions. Briefly, cells were treated with 0.3% Triton X-100 (10 min) and TUNEL reaction mixture (60 min) at 37 °C. Cell nuclei were stained with DAPI (1:1,000). Images were acquired with a fluorescence microscope, and apoptotic cells appears red.

## Immunofluorescence

Cells were placed on glass slides and fixed with 4% paraformaldehyde, blocked with bovine serum albumin (BSA) and incubated with primary antibodies overnight at 4 °C, followed by incubation with secondary antibodies. Nucleus was stained with DAPI (C1002, Beyotime Biotechnology, Shanghai, China) for 10 min at room temperature. Images were taken with a Zeiss LSM900 or a fluorescence microscope (AX10 VERT A1, Zeiss, Germany). The colocalization of ENO1 and YWHAZ analysis was calculated by ImageJ software.

## Immunoprecipitation and co-immunoprecipitation assays

The enriched proteins were incubated with 2 µg of antibodies (anti-acetyl lysine, anti-HA, anti-ENO1, anti-Flag, and anti-YWHAZ) and 30 µL of magnetic agarose beads were added to the mixture. The mixture was then incubated in a horizontal mixer at 4 °C overnight. Subsequently, the beads were heated at 100 °C for 5 min until proteins were separated from beads. The protein amounts were analysed by western blotting.

### Acetyl-Coenzyme A (CoA) measurement

The intracellular level of acetyl-CoA was quantified using the Elabscience Acetyl Coenzyme A ELISA Kit (E-EL-0125, Wuhan, China). Cells were washed with cold PBS twice and the cell membrane was destructed after repeated freeze–thaw cycles. Then, the cells were centrifuged at 4 °C and 1,500 × g for 10 min. The supernatant was analyzed using an ELISA Kit to detect acetyl‐CoA levels according to the manufacturer’s instructions.

### Animal study

Nude mice (BALB/c) were housed in each cage in a specific pathogen-free room with a 12-hour light/dark schedule at 25 ℃±1 ℃ and were fed an autoclaved chow diet and water ad libitum. In subcutaneous implantation nude mice models, tumor growth was monitored every 2 days and tumor volumes were calculated using the formula (a × b^2^) / 2, where a and b represent the longest and shortest diameters of the tumors respectively. The entire tumors were fixed with 4% paraformaldehyde prior to dehydration and embedding in paraffin. Sections were stained with H&E following standard protocols and counted with a microscopic count assay. Survival was assessed using Kaplan-Meier curve analysis.

### Statistical analysis

Data were presented as means ± SEMs and the statistical analysis were performed with GraphPad Prism 9.0 software (GraphPad Software Inc., CA, USA). Unpaired Student’s t test was used to compare differences between two groups, while analysis of variance (ANOVA) was utilized for comparisons among multiple groups. Survival analysis was performed using the Kaplan–Meier method, with the log-rank test employed to assess statistical significance. A threshold of *P* < 0.05 was considered statistically significant.

## Results

### ENO1 is upregulated in MM and is correlated with poor prognosis

**Fig. 1:**
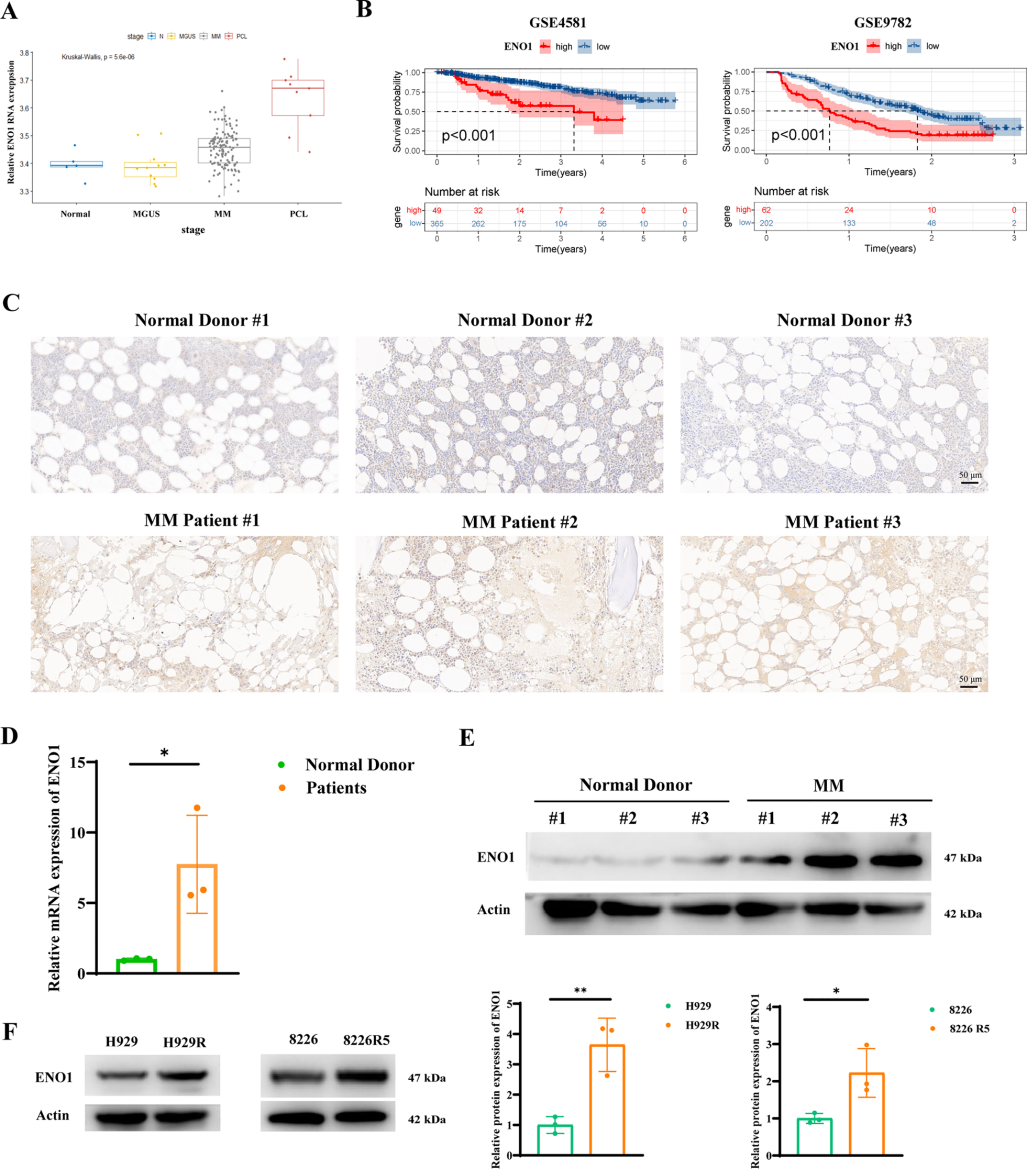
ENO1 is identified as a drug-resistance related gene and is associated with poor clinical prognosis in MM. **A** The expression levels of ENO1 at different MM stages are presented. **B** Kaplan-Meier analysis of the overall survival of MM patients with different ENO1 expression levels. **C** The expression of ENO1 in MM patients and normal donors were monitored by immunohistochemistry (IHC). Scale bar: 50 μm. **D** Total RNA was extracted from the bone marrow of MM patients and healthy donors, and detection of the relative ENO1 mRNA levels. **E** The expression level of ENO1 was detected by western blot (WB) in bone marrow from MM and normal tissues (N) from healthy donors. **F** WB analysis displaying ENO1 protein levels in susceptible MM strains (H929 and 8226) and corresponding bortezomib-resistant strains (H929R and 8226R5). *P < 0.05, **P < 0.01, ***P < 0.001

Bioinformatics analysis revealed that the RNA level of ENO1 was associated with disease progression (Fig. 1A). Moreover, overall survival was worse in the high ENO1 gene signature group than in the low ENO1 gene signature group, as shown in the GSE4581 and GSE9782 datasets (Fig. 1B). ENO1 expression was evaluated in samples from 3 patients with MM collected at Shanghai East Hospital, Tongji University School of Medicine, using IHC analysis, qRT-PCR, and western blotting. The IHC results indicated that ENO1 was markedly upregulated in the MM bone marrow (BM) (Fig. 1C). Additionally, qRT-PCR and western blot analysis verified the high expression of ENO1 in MM BM (Fig. 1D and E). Additionally, immunoblotting analysis revealed increased expression of ENO1 in BTZ-resistant MM cell lines (Fig. 1F). Taken together, these findings revealed a strong correlation between ENO1 expression and progression and poor survival. In addition, immunoblotting analysis indicated increased expression of ENO1 in drug-resistant MM cell lines.

### ENO1 knockdown causes cell cycle arrest and suppresses tumorigenicity

**Fig. 2:**
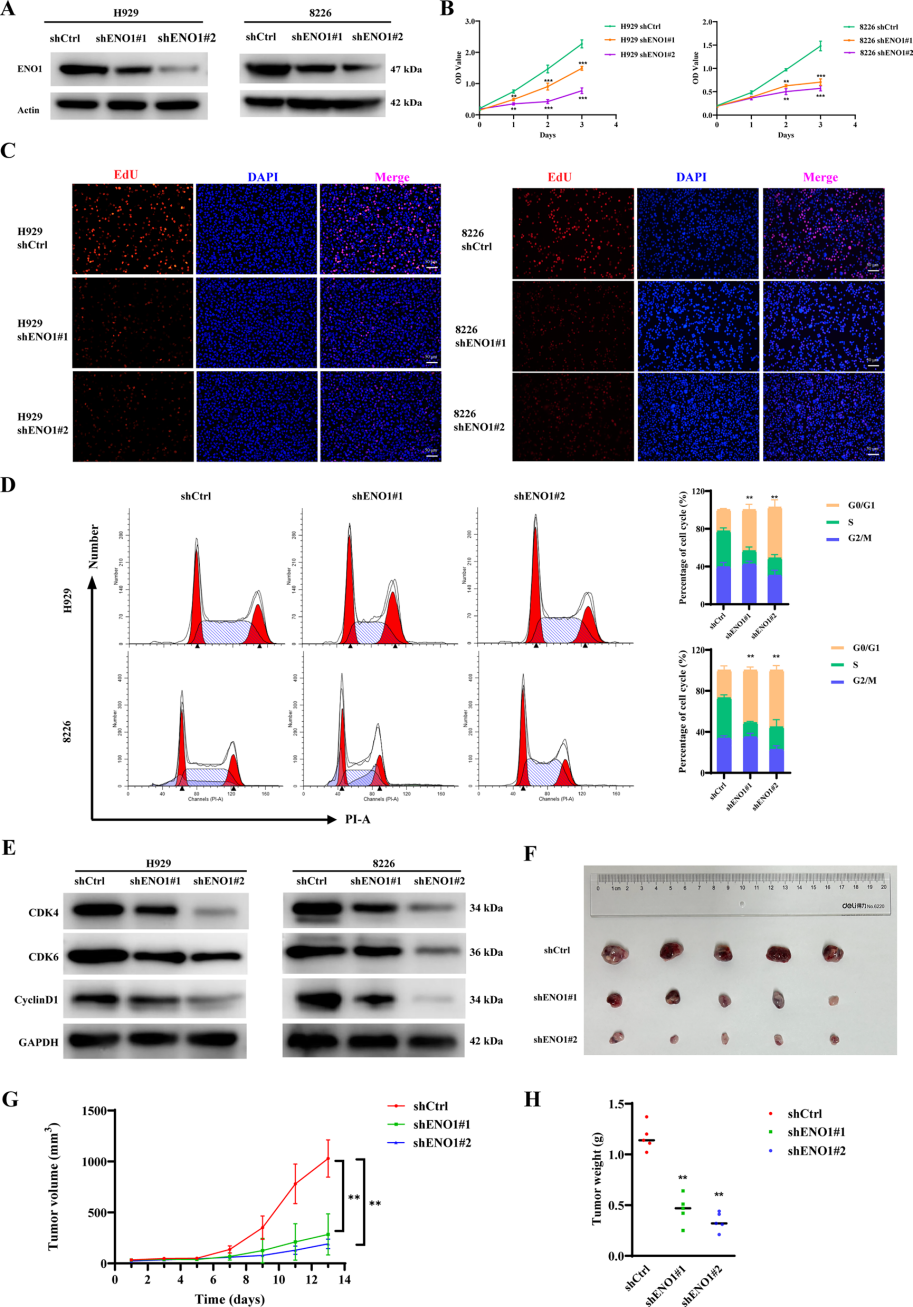
Knockdown of ENO1 suppresses MM proliferation. **A** The protein levels of ENO1 in the stably transformed ENO1 knockdown strain. **B** Cell survival of ENO1 knockdown and corresponding control MM cells, as determined by CCK8 assay. **C** EdU incorporation assay (scale bar: 50 μm) of ENO1 knockdown MM cells and the corresponding control cells. **D** Cell cycle analysis of MM cells stably transfected with shENO1 or shCtrl were pre-treated with serum-free medium for 24 h. The data are shown as means ± SDs with *P* values based on unpaired *t* test. **E** Immunoblotting analysis of G0/G1 phase-related protein (CDK4, CyclinD1 and CDK6) expression in MM cells stably transfected with shENO1 or shCtrl. Actin served as a loading control. **F** Differences in tumor size between different groups of nude mice after subcutaneous injection of shENO1 or shCtrl cells. **G–H** The growth curve and weight of xenograft were obtained in nude mice using stably expressing shENO1 or shCtrl cells. *P < 0.05, **P < 0.01, ***P < 0.001

To study the biological functions of ENO1 in MM, H929 and 8226 cells were infected with ENO1 short hairpin RNA (shRNA). Two specific shRNAs were designed to establish ENO1 knockdown models, and their knockdown efficiencies were validated through qRT-PCR (Figure S1A) and western blotting (Fig. 2A). To investigate the impact of ENO1 on cell proliferation, CCK-8 and EdU assays were conducted, revealing that ENO1 knockdown significantly attenuated the growth of MM cells (Fig. 2B and C). Subsequently, the influence of ENO1 on the cell cycle was examined, and the results indicated that ENO1 knockdown led to significant arrest of cells in the G0/G1 phase (Fig. 2D). Furthermore, ENO1 knockdown reduced the levels of G0/G1 phase-related proteins (cyclinD1, CDK4, and CDK6) (Fig. 2E). To investigate the biological roles of ENO1 in vivo, we established xenograft models using ENO1 knockdown cell lines. The growth (Fig. 2F-G) and weight (Fig. 2H) of xenografts was significantly decreased in ENO1-KD group compared with that in control group. Moreover, to further examine the effect of ENO1 knockdown in vivo, the immunohistochemistry (IHC) analysis indicated that the expression of Ki67 is much lower in ENO1-KD group compared to those in the control group (Figure S1D). These results suggest that knockdown of ENO1 exerts a significant inhibitory effect on MM proliferation and tumorigenicity.

### ENO1 knockdown in MM cells notably enhances sensitivity to bortezomib

**Fig. 3:**
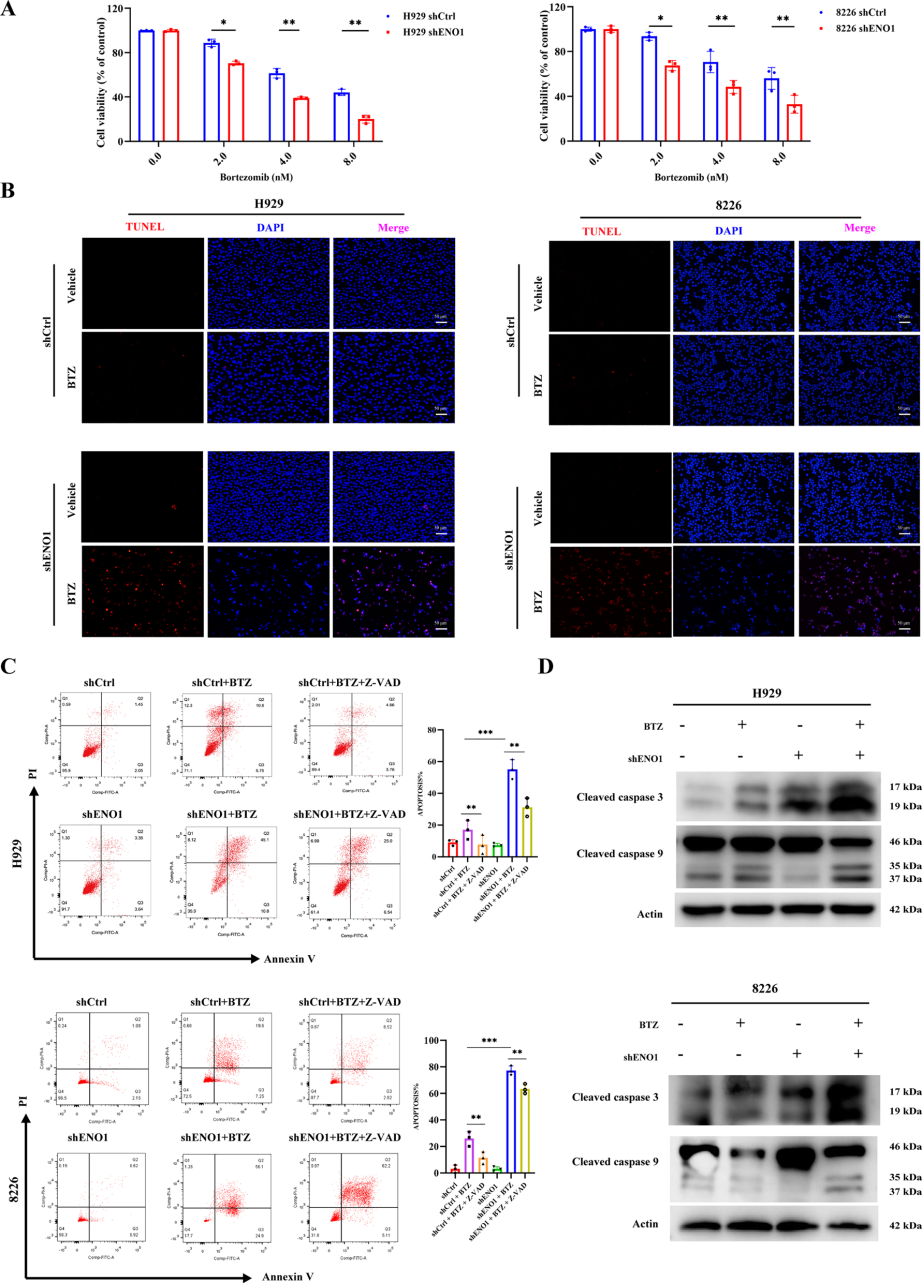
ENO1 knockdown increased the sensitivity of MM cells to BTZ. **A** CCK-8 assay was performed to evaluate cell viability after treatment with different concentrations of BTZ for 72 h in shCtrl and shENO1 cells. **B** Evaluation of cell apoptosis through TUNEL assay (scale bar: 50 μm) after treatment with vehicle or BTZ in shCtrl and shENO1 cells. **C** Evaluation of cell apoptosis after treatment with vehicle, BTZ, and Z-VAD, in MM cells. **D** Apoptosis-related protein levels were measured by immunoblotting after treatment with vehicle or BTZ in shCtrl and shENO1 cells. *P < 0.05, **P < 0.01, ***P < 0.001

The function of ENO1 in conferring resistance to BTZ was first explored. shENO1#2, which had a higher knockdown efficiency, was selected for subsequent experiments. Knockdown of ENO1 enhanced the cellular response to BTZ in MM cells (Fig. 3A). Additionally, TUNEL assays demonstrated that compared with control treatment, knockdown of ENO1 resulted in a greater proportion of apoptotic cells when the cells were exposed to the same concentration of BTZ (Fig. 3B). Notably, cell flow cytometry similarly revealed that knockdown of ENO1 increased the sensitivity of MM cells to BTZ, and the percentage of apoptotic cells was elevated, which could be partially rescued by a pan-apoptotic inhibitor (Z-VAD-FMK) (Fig. 3C). Furthermore, compared with control treatment, we found that BTZ treatment increased the activation of apoptosis-related proteins, such as cleaved caspase-3 and cleaved caspase-9 in ENO1-KD MM cells (Fig. 3D).

### ENO1 inhibitor notably synergizes with bortezomib Both in vitro and in vivo

**Fig. 4:**
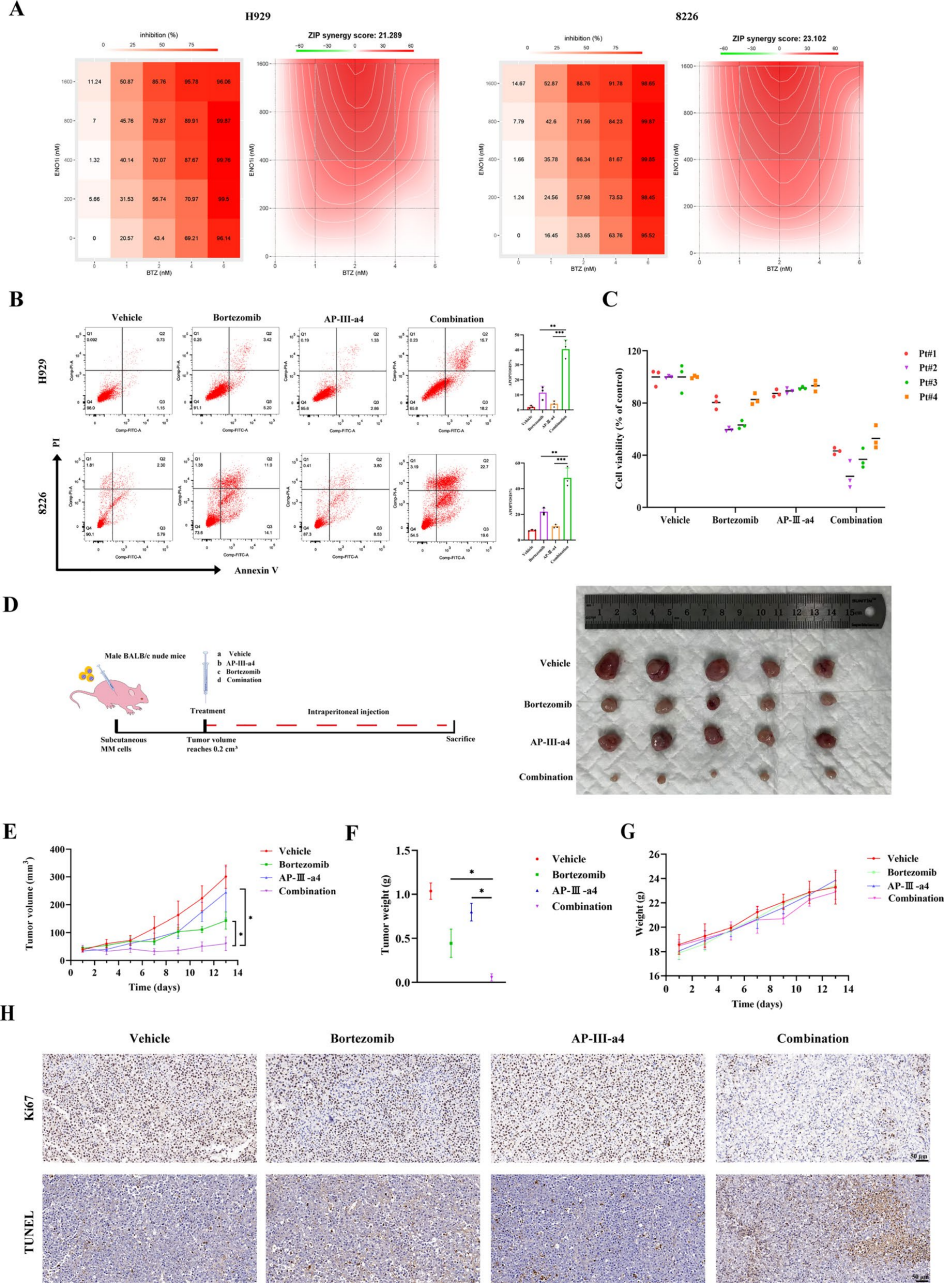
Inhibitory effects of combination therapy with ENO1i and BTZ. **A** H929 and 8226 cells were cotreated with the indicated concentrations of ENOi (AP-III-a4, 0.2–1.6 µM) and various concentrations of BTZ (1–6 µM) either alone or in combination for 72 h. Cell viability was assessed using a CCK-8 assay. Dose-response matrix (inhibition) for ENOi and BTZ. The inhibition ratio is positively related to the degree of red. The drug interaction landscapes are based on the ZIP model. **B** Flow cytometry was used to detect the cell apoptosis of MM cells treated with ENO1i (AP-III-a4) and BTZ. **C** The viability of cells from MM patients were evaluated after ENO1i (AP-III-a4), BTZ, and combination treatment for 72 h. Pt# represents patient number. Pt#1 and Pt#4 were diagnosed with relapsed/refractory multiple myeloma (RRMM). Data are presented as the means ± SD of three independent experiments. **D** The medication process is shown above via a schematic diagram of passage. MM subcutaneous tumor-bearing mice were treated with either ENO1i (10 mg/kg), BTZ (1 mg/kg) or a combination of both. Normal saline was used as a control. Representative images of the tumours from each group (*n* = 5 mice/group). **E** Tumour sizes were measured every 2 days. Tumour volumes are displayed through the growth curve.** F** Tumour weights were detected in different treatment groups. **G** Averages and SDs of mice weights versus the time (mean weight SD). **H** Ki-67, TUNEL staining of the vehicle, ENO1i (AP-III-a4), BTZ, and combination treated xenograft tumour tissues (original magnification: ×400). *P < 0.05, **P < 0.01 and ***P < 0.001 versus the control group

To further evaluate the potential efficacy of the ENO1 inhibitor in the treatment of MM, we detected whether the ENO1 inhibitor could synergize with BTZ. AP-III-a4 is a well-known inhibitor of ENO1 [8]. Simultaneous administration of ENO1i and BTZ to MM cells led to more potent cytotoxic effects (Fig. 4A, left panel) and significantly higher levels of apoptosis (Fig. 4B) than that observed upon independent treatment with either ENO1i or BTZ. Additionally, dose-effect analysis revealed a synergistic cytotoxic effect of ENO1i and BTZ, with a ZIP synergy score > 10 in MM cells (Fig. 4A, right panel). Notably, two of the four patients with MM had newly diagnosed and untreated MM, and two patients had relapsed MM resistant to BTZ and dexamethasone therapy; we found that ENO1i also synergized with bortezomib in these clinical samples (Fig. 4C). Additionally, we constructed an MM xenograft model, and the combination of ENO1i and BTZ led to a more pronounced growth inhibitory effects than that observed either ENO1i or BTZ alone in vivo (Fig. 4D and E). Much lower tumour weights were also observed in the combination group (Fig. 4F). Moreover, during the treatment period, there were no significant differences observed in the body weights of the mice among the various groups (Fig. 4G). The expression of Ki-67 in the tumour xenografts was evaluated by IHC and the synergistic effect of the two drugs was also assessed by the TUNEL assay. The findings suggested that co-administration of ENOi and BTZ led to a decrease in Ki-67 and an increase in apoptosis compared to each drug alone (Fig. 4H). Taken together, the present findings suggest that the ENO1 inhibitor potentiates the antitumour activity of BTZ, providing novel ideas for targeted drug development.

### ENO1 induces mitophagy in MM cells

**Fig. 5:**
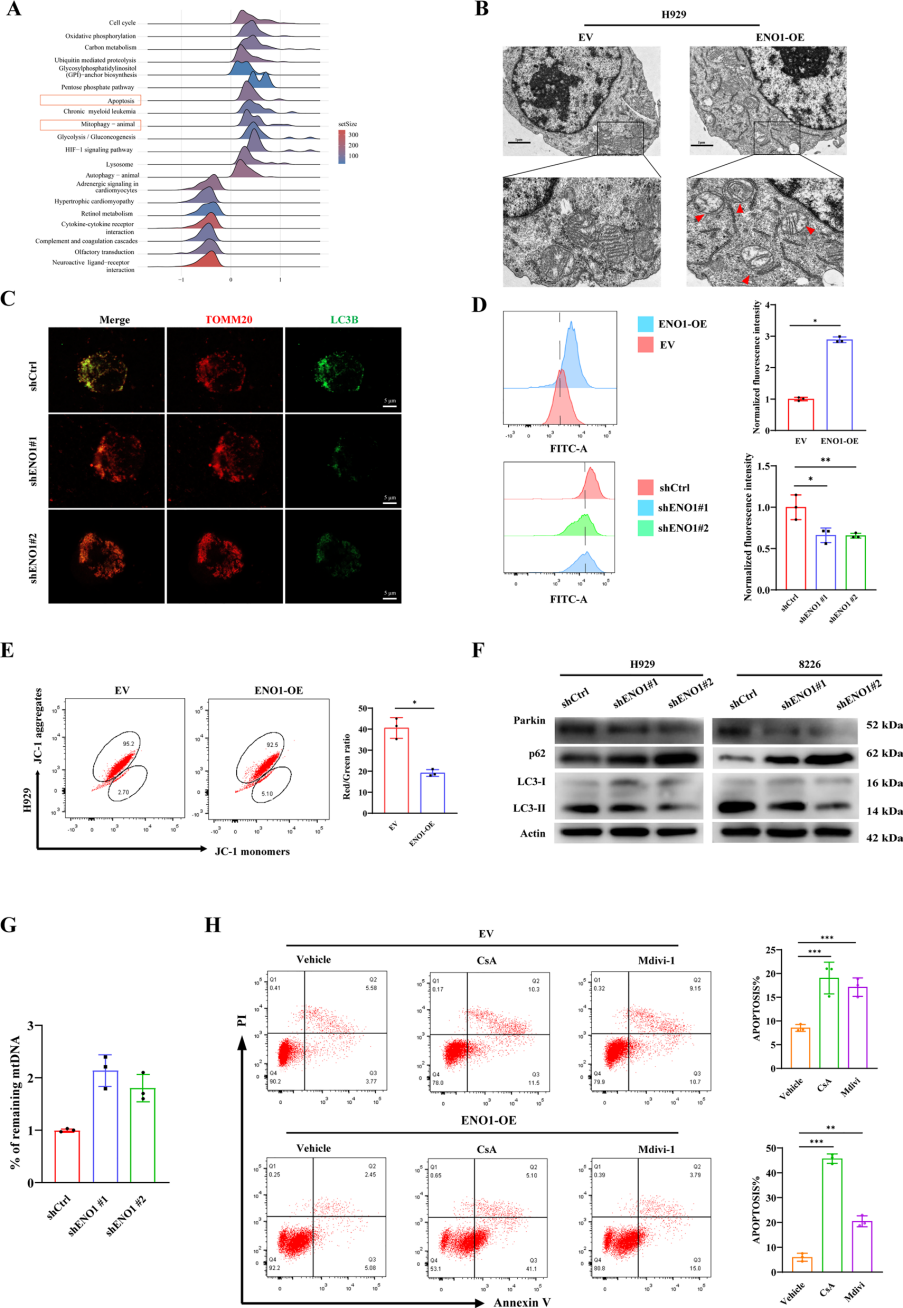
ENO1 promotes mitophagy in MM cells. **A** Ridge plot of KEGG pathway enrichment analysis for differentially expressed genes in MM patients stratified by ENO1 high- and low-expression levels. The horizontal axis represents the NES, and all results are statistically significant with *P* < 0.05. **B** Representative TEM images depicted the ultrastructure of H929 vector- or ENO1-overexpressing cells. Red arrows indicate autophagic vacuoles. (Scale bars: 1 μm). **C** The colocalization of mitochondria and autophagosomes was determined by immunofluorescence staining. Scale bar:5 μm. **D** H929 ENO1-OE cells, shENO1 cells, and their respective corresponding control cells were stained with Fluo-4 AM calcium probe and detected using flow cytometry. **E** JC-1 staining characterized by the transformation of red fluorescence to green fluorescence was used to measure the MMP after treatment of transfected with vector plasmid or ENO1-OE plasmid. **F** Western blot analysis of Parkin, p62, and LC3B protein levels in H929 and 8226 cells with or without ENO1 knockdown after treatment with CCCP. **G** Mitochondria DNA content was measured by qRT-PCR in the indicated cells. **H** Flow cytometry was used to detect the cell apoptosis of MM cells treated with CsA (5 µM) or Mdivi-1 (20 µM) for 24 h. *P < 0.05, **P < 0.01, ***P < 0.001

To decipher the potential mechanism by which ENO1 influences MM growth and progression, we queried a public database (GSE24080) for enrichment of ENO1-related differential genes. The differentially expressed genes were notably enriched in biological processes, including apoptosis and mitophagy (Fig. 5A). It has been reported that ENO1 maintains mitochondrial membrane stability in the cytoplasm [27]. Accordingly, we further investigated whether it was associated with mitophagy. Transmission electron microscopy (TEM) revealed an increase in the presence of bilayer membrane-bound autophagosomes in MM cells exhibiting high ENO1 expression, thereby corroborating the robust association between ENO1 and mitophagy (Fig. 5B). A hallmark of autophagy activation is the lipidation of LC3B I (cytosolic microtubule-associated protein 1 A/1B light chain 3B), which occurs when it binds to phosphatidylethanolamine to form LC3B II. Therefore, we detected less diffuse fluorescence of LC3B localized to mitochondria using immunofluorescence after ENO1 knockdown in the presence of mitophagy activator CCCP (Fig. 5C). When mitophagy occurs, the cytoplasmic calcium ion concentration is elevated due to mitochondria dysfunction. Using a Fluo-4 AM calcium probe, we found that ENO1 overexpression resulted in increased cytoplasmic calcium ion concentrations while knockdown of ENO1 had the opposite result (Fig. 5D). The JC-1 staining results demonstrated that ENO1 overexpression led to an attenuated MMP in MM cells (Fig. 5E), while knockdown of ENO1 can enhance MMP (Figure S2B). Western blot analysis ulteriorly demonstrated that ENO1-KD in MM cells resulted in weakened LC3B lipidation after CCCP treatment (Fig. 5F). Mitophagy-related markers such as Parkin, LC3B, and p62 were also found to be decreased after ENO1 knockdown (Fig. 5F). Compromised mitophagy can alter the integrity of mitochondrial DNA (mtDNA) and result in the accumulation of damaged mtDNA [28]. ENO1-KD MM cells were treated with 10 µM oligomycin, a mitochondrial uncoupler that induces mitophagy through inhibiting complex III of the electron transport system. Consistently, qPCR analysis demonstrated a notable elevation of mtDNA in ENO1-KD cells compared to the corresponding control cells (Fig. 5G). To address the roles of ENO1-dependent mitophagy on cell growth and apoptosis, we treated ENO1 overexpression cells with CsA and Midvi-1, two mitophagy inhibitors that act via different mechanisms. Our results demonstrate that inhibiting mitophagy effectively reduces the proliferative advantage induced by ENO1 overexpression (Figure S2C) and eliminate the apoptosis escape caused by ENO1 overexpression (Fig. 5F). Collectively, these results indicated the important roles of ENO1 in mitophagy, which might closely correspond to MM proliferation and drug resistance.

### ENO1 interacts with YWHAZ and regulates YWHAZ protein stability

**Fig. 6:**
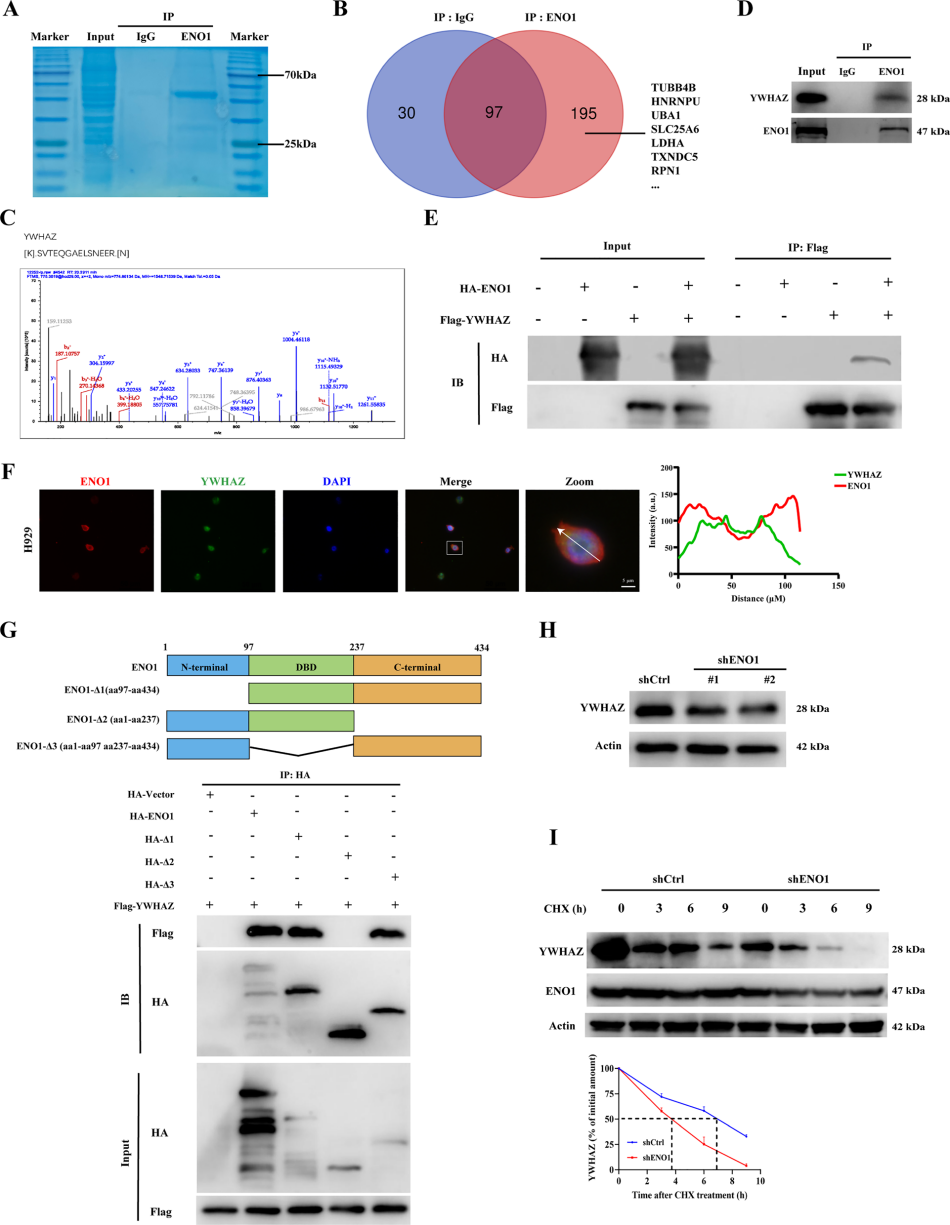
ENO1 interacts with YWHAZ and promotes its protein stability. **A** Total cell lysate was extracted from H929 cells, purified and resolved on SDS-PAGE. Coomassie brilliant blue stained gel exhibited differential bands, and the bands were retrieved and analysed by mass spectrometry. **B** Venn diagram showed that 195 proteins specifically pulled down from the IP group, excluding the 97 proteins that co-crossed between the IgG and IP groups. **C** Identified YWHAZ peptides are shown. **D** Endogenous YWHAZ in MM cells was immunoprecipitated using anti-ENO1 antibody with rabbit IgG as a nonspecific control. **E** HEK293T cells were transfected with Flag-YWHAZ, HA-ENO1, or both constructs. Cell lysates were immunoprecipitated using anti-Flag and the immunoprecipitants or input were analysed by immunoblotting with anti-HA or anti-Flag. **F** Co-localization between YWHAZ (green) and ENO1 (red) was analysed by confocal microscopy in H929 cells. (Scale bar: 5 μm). **G** Co-immunoprecipitation was conducted to investigate the interaction between YWHAZ and HA-vector, full‐length HA‐ENO1, and truncated forms of HA‐ENO1 in HEK293T cells. **H** In the case of ENO1 knockdown, YWHAZ and ENO1 protein levels were analysed by Western blot. Actin was used as the loading controls. **I** Downregulation of ENO1 increases YWHAZ degradation. Western blot detected the alteration of YWHAZ in H929 cells with treatment of 10 µg/mL CHX for the indicated times. Actin was used as a loading control

To identify the proteins that potentially interact with ENO1 and molecular functions, we performed a co-IP mass spectrometry (co-IP-MS) assay (Fig. 6A). This analysis identified 125 and 295 proteins in the IP-IgG and IP-ENO1 groups, respectively. A Venn diagram revealed that there were 195 proteins for the IP-ENO1 group-specific proteins (Fig. 6B). Next, the mass spectrometry result indicated that YHWAZ could interact with ENO1 (Fig. 6C), corroborated by database analysis showing a positive correlation between ENO1 and YWHAZ (Figure S3A). We next performed co-IP to verify the interaction between ENO1 and YWHAZ. In MM cells, YWHAZ was precipitated by the ENO1 antibody but not by the control IgG antibody (Fig. 6D). Further confirmation was achieved through reverse validation of the interaction between endogenous ENO1 and YWHAZ (Figure S3B). Additionally, an exogenous co-IP assay was conducted in HEK293T cells transfected with HA-tagged ENO1 (HA-ENO1), Flag-YWHAZ, or both, demonstrated an interaction between YWHAZ and ENO1 (Fig. 6E). Furthermore, confocal laser microscopy was employed to visualize the intracellular distribution of ENO1 and YWHAZ, which indicated a co-localization between YWHAZ and ENO1 in MM cells (Fig. 6F). To delineate the specific interaction domains between YWHAZ and ENO1, co-IP analyses were tested by truncating ENO1 into three segments based on the functional structure of the ENO1. Then we found that only the C-terminal deletion truncated mutants (HA-ENO1-237–434) could not bind to Flag- YWHAZ but not the N-terminal deletion mutant (HA-ENO1-1–97) and the DNA binging domain deletion mutant (HA-ENO1-97–237) (Fig. 6G). Next, we investigated the impact of ENO1 knockdown on the expression of YWHAZ and discovered that ENO1 had no effect on the RNA level of YWHAZ (Figure S3C), but significantly reduced the protein level of YWHAZ (Fig. 6H), suggesting that ENO1 modulates the YWHAZ expression through PTM. To substantiate the impact of ENO1 on YWHAZ protein stability, cycloheximide (CHX) chase assays were performed, revealing a marked decrease in YWHAZ protein stability following ENO1 knockdown (Fig. 6I).

### Acetylation-mediated proteasomal degradation is critical for the regulation of YWHAZ by ENO1

**Fig. 7:**
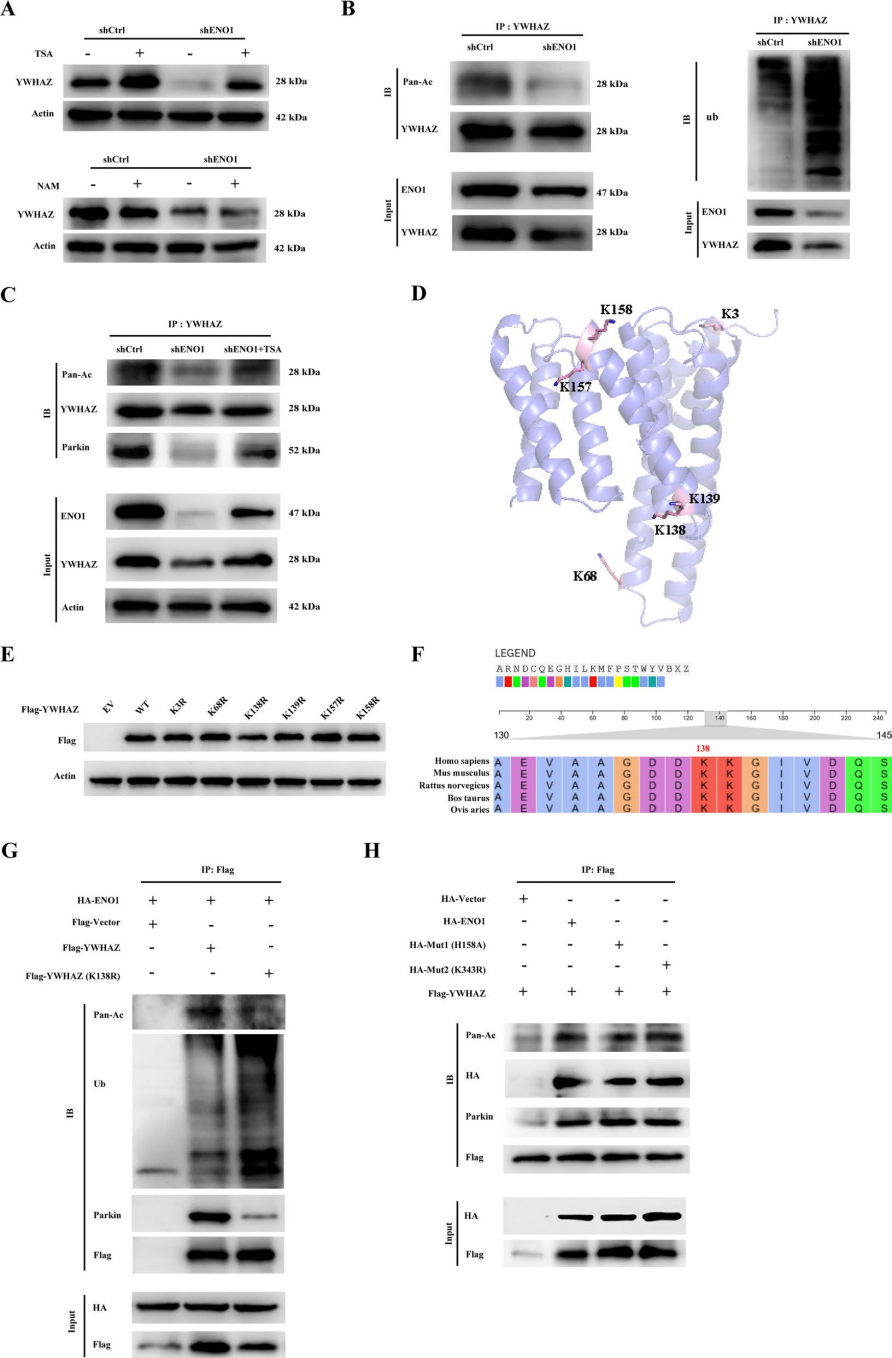
Acetylate on K138 modulates YWHAZ stability. **A** Western blot analysis of the alteration of YWHAZ in H929 cells with treatment of 10 µM (TSA) or 100 µM (NAM) for 8 h. Actin was used as a loading control. **B** HEK293T cells were transiently transfected with shCtrl or shENO1 plasmid. Immunoprecipitation was performed with anti-YWHAZ beads and the level of acetylation (Pan-Ac) and ubiquitination (ub) were detected with the corresponding antibodies. **C** Western blot of anti-YWHAZ immunoprecipitates from HEK293T cells with shCtrl or shENO1 transfection treated with/without TSA. **D** Diagram showing the sequence of acetylation sites of YWHAZ. **E** Western blot analysis of Flag-YWHAZ in lysates from HEK293T cells transfected with indicated Flag‐YWHAZ mutations. **F** Sequence alignment comparing the differences in the amino acids of YWHAZ in five species. **G** Western blot analysis of acetylation and ubiquitination of YWHAZ from HEK293T cells transfected with Flag-YWHAZ‐vector, WT, and K138R. **H** HEK293T cells were transfected with Flag-YWHAZ, HA-vector, HA-ENO1, HA-Mut1 (K158A), and HA-Mut2 (K343R) and detected with anti-HA, anti-Flag, anti-Parkin, and anti-Ace by Western blot analysis

Previous studies have demonstrated that the balance between acetylation and ubiquitination affects protein stability, and both modifications are present in YWHAZ [29]. We treated ENO1 knockdown cells and corresponding control cells with SIRT family inhibitors (NAM) and HDAC family inhibitors (TSA), and found that YWHAZ was pronouncedly more affected by the HDAC family inhibitor (Fig. 7A). We performed immunoprecipitation of overexpressed Flag-tagged YWHAZ from HEK 293T cells with or without ENO1 knockdown, and found that YWHAZ acetylation was present but weakened after ENO1 knockdown (Fig. 7B). Meanwhile, the level of YWHAZ ubiquitination increased after ENO1 knockdown (Fig. 7B). To further elucidate the relationship between YWHAZ acetylation and proteasomal degradation, we pretreated 293T cells with MG132 and subsequently examined YWHAZ acetylation via IP-western blot analysis. The results clearly showed that ENO1 knockdown attenuated YWHAZ acetylation, which could be reverted by HDACs inhibitor treatment (Fig. 7C). We immunopurified endogenous YWHAZ in ENO1 overexpression MM cells and then performed mass spectrometry analysis and six acetylated sites were identified (Fig. 7D). To elucidate the effect of these acetylating sites, we constructed six YWHAZ deacetylation-mimetic mutants (with Lys residues replaced by Arg residues) as follows: K3R, K68R, K138R, K139R, K157R, and K158R. Notably, the expression of the K138R YWHAZ mutant was significantly decreased compared with WT and other mutants (Fig. 7E). Multiple sequence alignment revealed that Lys138 was highly conserved among species (Fig. 7F). To explore the relationship between acetylation and ubiquitination, we overexpressed HA-ENO1 with vector, WT, and K138R YWHAZ. IP-western blot analysis indicated that the ubiquitination level of K138R was much lower than that of WT YWHAZ (Fig. 7G), suggesting that Lys138 is a crucial acetylation site for YWHAZ ubiquitination. Furthermore, the ability of YWHAZ to bind to parkin is also affected by Lys138 acetylation (Fig. 7G). Taken together, these results suggest that acetylation at Lys138 attenuates YWHAZ degradation via the ubiquitin‐proteasome pathway. To investigate whether ENO1 mediated YWHAZ degradation depended on its enzymatic activity, we constructed the enzymic inactive mutant of ENO1 [30]. Collectively, our results revealed that endogenous YWHAZ can interact with HA-ENO1 as well as its enzymatically mutant H158A and K343R, and the level of YWHAZ is not influenced by ENO1 enzyme activity (Fig. 7H). These results showed that neither the expression of YWHAZ nor its binding to ENO1 is dependent of ENO1 enzymatic activity.

### HDAC6 is the deacetylase of YWHAZ and the acetylation levels of YWHAZ is required for mitophagy

**Fig. 8:**
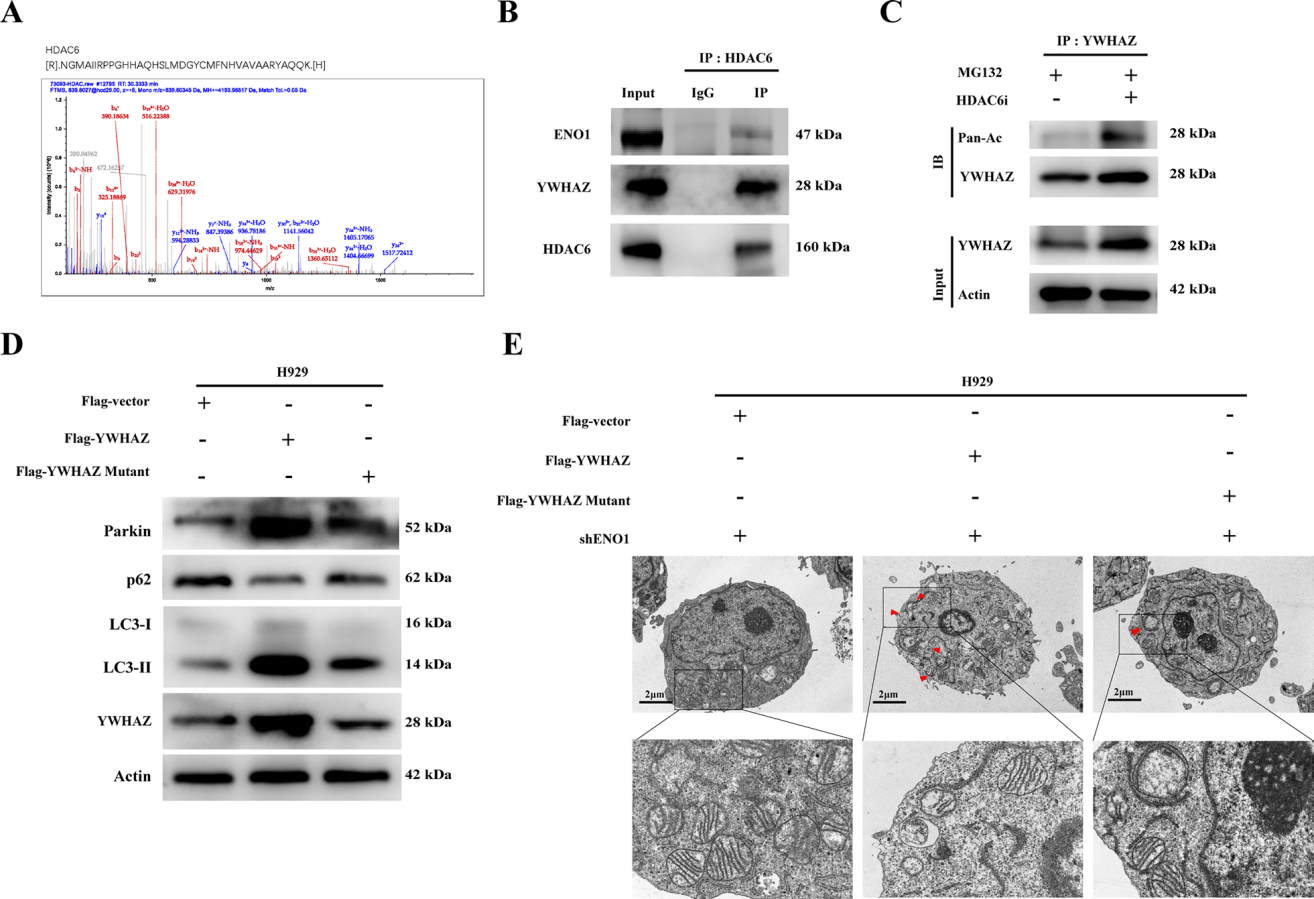
YWHAZ is acetylated by HDAC6 and the acetylation level of YWHAZ affects mitophagy. **A** Identified HDAC6 peptides are shown. **B** Immunoblot of anti-HDAC6 immunoprecipitates from H929 cells. **C** Immunoblot of cell lysates or anti-YWHAZ immunoprecipitates from H929 cells with vehicle or HDAC6i treated with MG132 (10 µM) for 2 h. **D** Immunoblot of Flag-YWHAZ from HEK293T cells transfected with Flag‐vector, Flag‐YWHAZ, and K138R. **E** Representative TEM images depicted the mitochondria ultrastructure of vector-, YWHAZ-OE, YWHAZ-mutant cells. Red arrows indicate autophagic vacuoles. (Scale bars: 2 μm)

Previous studies have showed that ENO1 functions as an HDAC-binding protein [31] and specifically correlates with HDAC6 [8]. In light of these findings, we conducted a co-IP mass spectrometry assay and identified HDAC6 as a binding partner (Fig. 8A). Our investigations subsequently centered on HDAC6, and found that HDAC6 interacts with YWHAZ in MM via co-IP assay (Fig. 8B). To confirm whether YWHAZ undergoes acetylation, H929 cells were treated with the selective HDAC6 deacetylase inhibitor Tubastatin A HCl. Endogenous YWHAZ acetylation level was increased under Tubastatin A HCl treatment (Fig. 8C). To further explore the impact of YWHAZ acetylation levels on mitophagy, we observed that overexpression of YWHAZ activated the mitophagy. However, mutation of the YWHAZ acetylation site did not result in a significant increase in the mitophagy marker (Fig. 8D). Furthermore, TEM revealed an increase in the presence of bilayer membrane-bound autophagosomes in MM cells exhibiting high YWHAZ expression but not in YWHAZ mutant, which confirmed that the K138 site is specifically deacetylated, mediating YWHAZ expression and mitophagy (Fig. 8E).

### ENO1 promotes MM mitophagy and drug resistance via YWHAZ

**Fig. 9:**
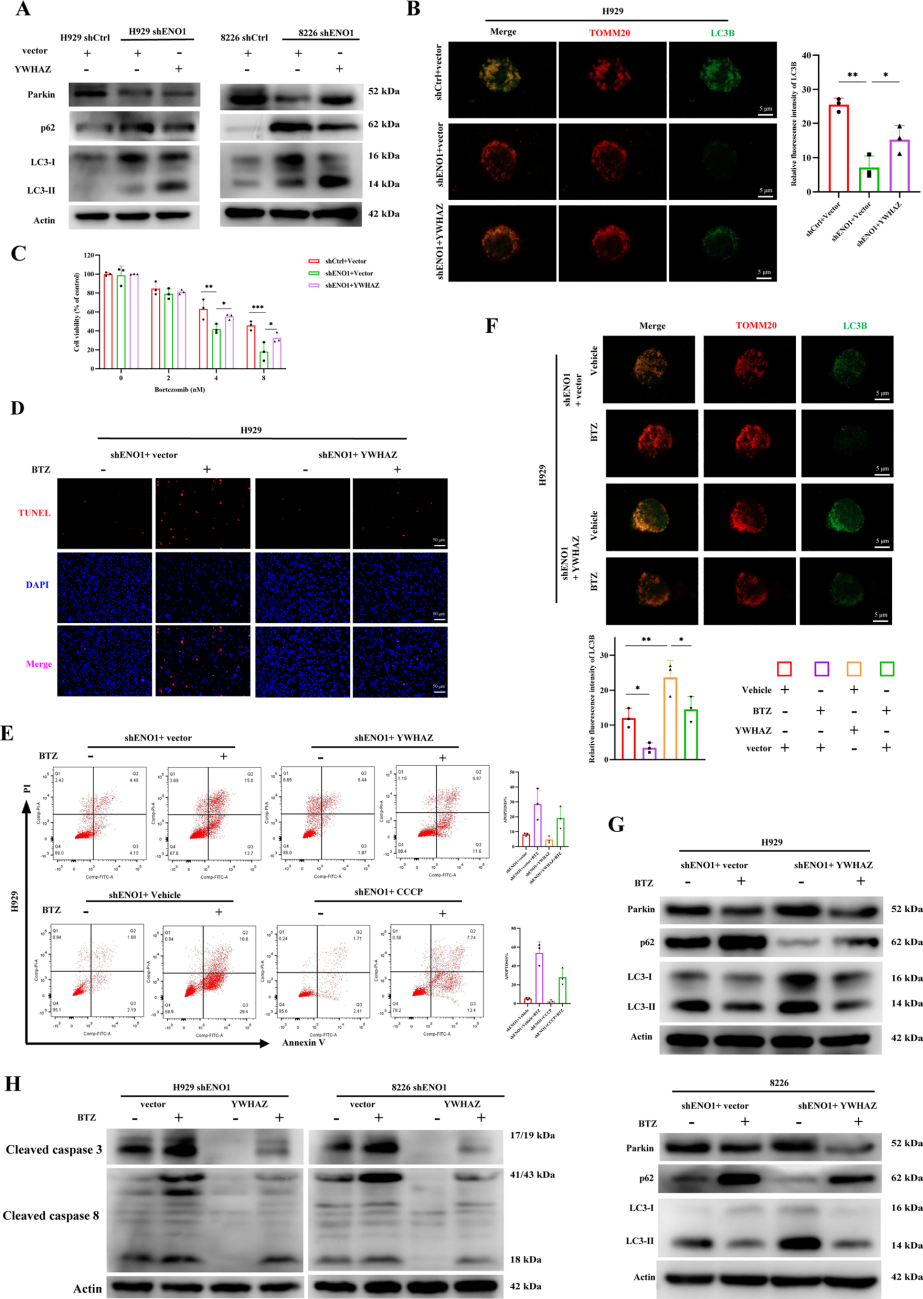
ENO1 regulates mitophagy through YWHAZ. **A** Western blot analysis of mitophagy-related proteins was detected in MM shCtrl and shENO1-derived cells treated with or without BTZ, and transiently transfected with or without vector or YWHAZ plasmid. **B** The co-localization between LC3B (green) and TOMM20 (red) was analysed by confocal microscopy in shCtrl and shENO1 cells transiently transfected with or without Vector or YWHAZ plasmid with CCCP (10 µM) stimulation. (Scale bar: 5 μm). **C** Cell viability was assessed after treatment of H929 shCtrl and shENO1-derived cells with different concentrations of BTZ and transiently transfected with or without vector or YWHAZ plasmid. **D** TUNEL assays of H929 shENO1 cells treated with or without BTZ and transiently transfected with or without vector or YWHAZ plasmid. **E** Apoptosis of shENO1 MM cells transiently transfected with vector or YWHAZ plasmid and treated with or without BTZ (upper panel). Apoptosis percentage of BTZ-treated shENO1 MM cells in the presence or absence of CCCP pretreatment with or without BTZ treatment (lower panel). **F** The co-localization between LC3B (green) and TOMM20 (red) was analysed by confocal microscopy in shCtrl and shENO1 cells transiently transfected with or without vector or YWHAZ plasmids with CCCP (10 µM) stimulation. **G** Western blot analysis of mitophagy-related proteins was detected in shCtrl and shENO1-derived cells treated with or without BTZ and transiently transfected with or without vector or YWHAZ plasmid. **H** Western blot analysis of apoptosis-related proteins was detected in shCtrl and shENO1-derived cells treated with or without BTZ and transiently transfected with or without vector or YWHAZ plasmid. *P < 0.05, **P < 0.01, ***P < 0.001

We validated the involvement of YWHAZ in the ENO1-mediated tumour-protective mitophagy and drug resistance, and performed rescue assays. Western blotting and immunofluorescence showed that the ectopic expression of YWHAZ reversed the suppressive effect of ENO1 knockdown on mitophagy (Fig. 9A and B). Additionally, the impact of YWHAZ overexpression on ENO1-mediated drug-resistance was explored. YWHAZ overexpression reversed the sensitivity of MM cells to BTZ caused by ENO1 silencing (Fig. 9C and D). We further explored whether ENO1-mediated resistance via YWHAZ results from the activation of mitophagy. BTZ treatment induces apoptosis and inhibits cytoprotective mitophagy [32, 33]. The findings revealed that YWHAZ overexpression diminished ENO1 knockdown-mediated resensitization to BTZ, as observed after treatment with mitophagy inducers (CCCP) (Fig. 9E and F), and the downregulation of mitophagy and apoptosis related markers (Fig. 9G and H). In summary, the results strongly indicate that YWHAZ promotes mitophagy and drug resistance by functioning as a downstream target of ENO1.

## Discussion

Proteasome inhibitors (PIs) have become the backbone therapy for MM. They have exhibited great efficacy in clinical applications. Nevertheless, despite their effectiveness, patients nonetheless develop resistance to this therapy.

There is mounting evidence indicates that mitophagy plays a pivotal role in both cancer growth and drug resistance. Mitophagy can act as a protective mechanism against apoptosis by removing damaged mitochondria, preventing the release of pro-apoptotic signals, and promoting cell survival.

It has been reported that PINK1-Parkin pathway-mediated mitophagy and mitochondrial damage promote chemotherapy resistance in small cell lung cancer (SCLC) patients [34]. In addition, mitophagy is activated in HCC (hepatocellular carcinoma) with mitochondrial dysfunction and helps HCC cells survive drug treatment or other stresses [35]. Furthermore, in breast cancer, targeting mitophagy can help overcoming chemoimmunotherapy resistance by redirecting PD-L1 to the mitochondria [36]. However, in MM, the exact mechanism by which mitophagy mediates drug resistance remains unclear. As a result, gaining a deeper understanding of the molecular mechanisms that underlie resistance to PIs and mitophagy is crucial for the development of effective clinical therapies for MM.

ENO1 has been extensively studied as a glycolytic enzyme [37], but a growing body of research has indicated that it is a multifunctional protein, possessing additional roles beyond its traditional metabolic activity. However, the underlying mechanisms and biological outcomes remain unelucidated. By analysing publicly available databases, we found that ENO1 was upregulated in BTZ-resistant MM cells compared to that in the corresponding sensitive cell lines. We found that MM patients with high ENO1 expression had a stronger malignant potential and poorer prognosis. In the current study, ENO1 knockdown suppresses tumorigenicity and induced MM cell cycle arrest. Moreover, ENO1 knockdown increased the sensitivity of MM to BTZ and elevated apoptosis. This process can be partially restored by pan-apoptosis inhibitor, indicating that cell death is multifaceted and not solely reliant on apoptosis; however, in our model, apoptosis is the primary mode of cell death. To further explore the value and significance of inhibiting ENO1 in clinical translation, we investigated whether the ENO1 inhibitor (AP-III-a4) could synergize with BTZ in MM cells, and found that co-administration had synergistic effects simultaneously in vitro and in vivo. Interestingly, it’s worth noting that this synergistic effect was effective in clinical samples from both newly diagnosed and relapsed patients with MM.

Mitophagy serves as a mechanism for mitochondrial quality control, eliminating dysfunctional and damaged mitochondria. Recent researches have pinpointed the crucial role of PINK1-Parkin mediated mitophagy. Parkin is an E3 ubiquitin ligase capable of self-ubiquitination, which tags dysfunctional mitochondria with ubiquitin chains to facilitate their recognition and subsequent degradation via mitophagy [38–40].

We found that the expression of Parkin and LC3B was downregulated in ENO1-depleted MM cells. Furthermore, we observed that mitochondrial homeostasis was disturbed when ENO1 knockdown; however, cells could not clear the impaired mitochondria eventually leading to apoptosis.

Moreover, we found that in MM cells overexpressing ENO1, the elevated level of mitophagy was dependent on ENO1 expression, which may play a role in the insensitivity of MM to BTZ. These data further illustrate that knockdown of ENO1 could lead to impaired mitophagy, preventing cells from clearing damaged mitochondria and driving apoptosis. ENO1-induced mitophagy decreases MMP loss. The rise in cytosolic calcium could result from the release of calcium from damaged mitochondria. At the same time, it may explain that ENO1-promoted apoptotic escape of MM cells may be due to subsequent mitochondrial adaptation resulting from increased clearance of damaged organelles.

Mechanistically, YWHAZ belongs to the 14-3-3 family, which has been reported to affect mitophagy by interacting with pivotal mitophagy-related proteins [15]. In the present study, we found that ENO1 promoted YWHAZ protein stability and triggered cytoprotective mitophagy by interacting with YWHAZ. It has been reported that acetylation and ubiquitination could compete for the same lysine site [41]. Our data showed that acetylation suppressed the ubiquitinated degradation of YWHAZ. Furthermore, we applied an LC–MS/MS assay and identified several acetylated lysine residues in YWHAZ. Among these sites, Lys138 is essential for YWHAZ protein stability, and an evolutionary conservation analysis and the effect of mutations of this site suggest its importance. HDAC6 is a key number of class IIa histone deacetylases and involved in the pathogenesis of various cancers [42]. Numerous studies have demonstrated that HDAC6 is essential in various biological processes by modulating PTM [43, 44]. In this study, we found that HDAC6 could bind to YWHAZ and ENO1, and inhibition of HDAC6 activity promoted the protein stability of YWHAZ by inhibiting its proteasomal degradation.

ENO1 activates mitophagy by increasing the acetylation level of YWHAZ, leading to a decrease in its ubiquitination level and the inability for YWHAZ to be degraded, an increase in the stability of YWHAZ and thus an increase in its binding to Parkin, which further inhibits the degradation of Parkin, leading to an increase in the ability of MM cells to clear damaged mitochondria. This promotes mitochondrial homeostasis in MM cells, which leads to apoptotic escape, and an insensitivity to pharmacological stresses such as BTZ. Overexpression of YWHAZ in ENO1-KD cells was found to revertively rescue increased sensitivity to BTZ. Taken together, we demonstrated for the first time that ENO1 is a key factor in the regulation of mitophagy via YWHAZ to promote MM resistance to apoptosis and drug insensitivity. ENO1 regulates the acetylation level of YWHAZ at the K138 site through HDAC6, thereby enhancing the stability of YWHAZ. Furthermore, we found that ENO1 inhibitors have synergistic effects with the first-line chemotherapeutic agents for MM both in vitro and in clinical samples, providing a theoretical basis for the development of new therapies for MM.

## Conclusion

Our study indicates that ENO1 plays a crucial role in the development and progression of MM. ENO1 enhances mitophagy through the stabilization of YWHAZ, which in turn inhibits apoptotic pathways and contributes to drug resistance. ENO1 promotes the acetylation of lysine at position 138 of YWHAZ, inhibiting its ubiquitination and degradation, thereby enhancing YWHAZ stability. Targeting ENO1 could offer a promising approach to overcome the resistance of MM to BTZ and improve therapeutic outcomes.

## Supplementary Information

Supplementary Material 1.

Supplementary Material 2.

Supplementary Material 3.

Supplementary Material 4.

Supplementary Material 5.

## Data Availability

The dataset(s) supporting the findings of this study are included within the article.
