# Supplementary figures and images for: Targeting enolase 1 reverses bortezomib resistance in multiple myeloma through YWHAZ/Parkin axis
Source: J Biomed Sci. 2025 Jan 20;32:9. doi: 10.1186/s12929-024-01101-x (PMC11744840; doi:10.1186/s12929-024-01101-x)

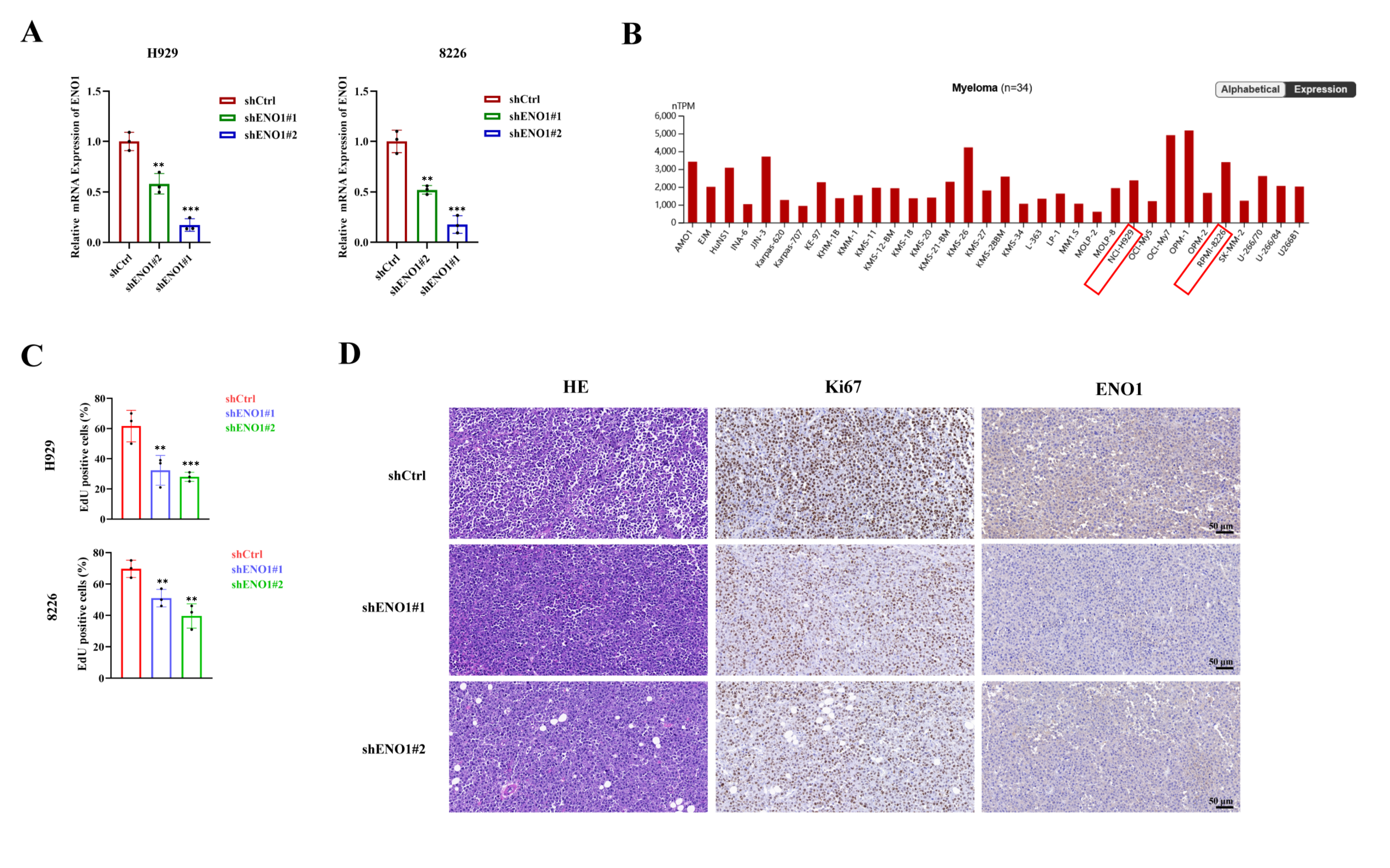

Supplement: Supplementary file 1 — Supplementary Material 1. [file 12929_2024_1101_MOESM1_ESM.tif]

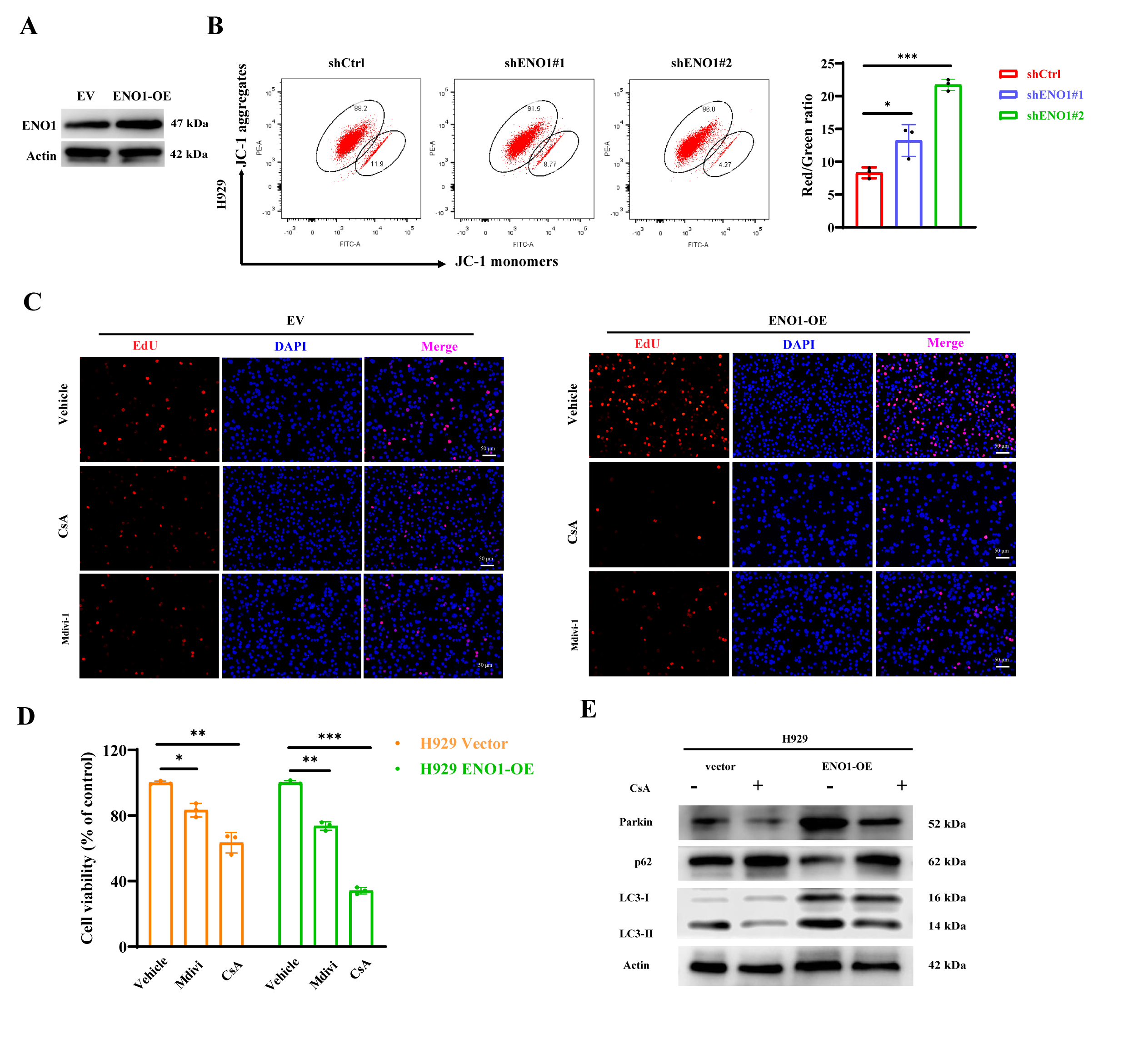

Supplement: Supplementary file 2 — Supplementary Material 2. [file 12929_2024_1101_MOESM2_ESM.tif]

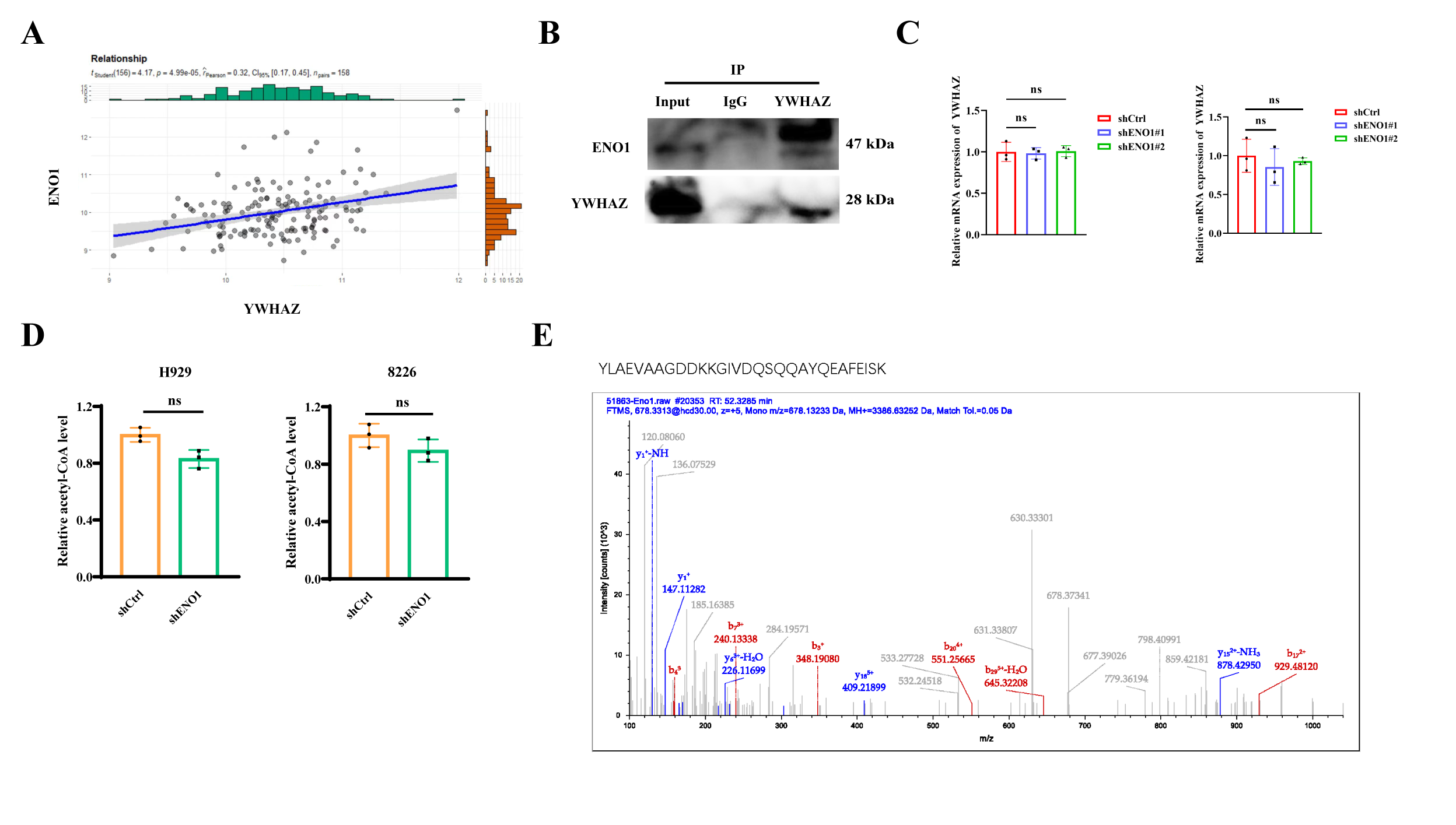

Supplement: Supplementary file 3 — Supplementary Material 3. [file 12929_2024_1101_MOESM3_ESM.tif]
