# Supplementary material for: Targeting enolase 1 reverses bortezomib resistance in multiple myeloma through YWHAZ/Parkin axis
Source: J Biomed Sci. 2025 Jan 20;32:9. doi: 10.1186/s12929-024-01101-x (PMC11744840; doi:10.1186/s12929-024-01101-x)
Supplement: Supplementary file 4 — Supplementary Material 4. [file 12929_2024_1101_MOESM4_ESM.docx]

**Supplementary Table 1**

| **Supplemental Table 1. The sequences of primers used in qRT-PCR** | |
| --- | --- |
| **Gene** | **Sequences (5′-3′)** |
| ENO1 | Forward: AAAGCTGGTGCCGTTGAGAA |
|  | Reverse: GGTTGTGGTAAACCTCTGCTC |
| YWHAZ | Forward: TGTAGGAGCCCGTAGGTCATC |
|  | Reverse: GTGAAGCATTGGGGATCAAGA |
| DLOOP | Forward: ACCTGTGATCCATCGTGATGT |
|  | Reverse: GCCCATAACACTTGGGGGTA |
| 12S | Forward: TAGCCCTAAACCTCAACAGT |
|  | Reverse: TGCGCTTACTTTGTAGCCTTCAT |
| ACTB | Forward: CATGTACGTTGCTATCCAGGC |
|  | Reverse: CTCCTTAATGTCACGCACGAT |

**Supplementary Table 2**

| **Supplemental Table 2. The sequences of primers used in ENO1 knocked down** | |
| --- | --- |
| **Gene** | **Sequences (5′-3′)** |
| shCtrl | T: GATCTGTTCTCCGAACGTGTCACGTTTCAAGAGAACGTGACACG  TTCGGAGAATTTTTTC |
|  | B: AATTGAAAAAATTCTCCGAACGTGTCACGTTCTCTTGAAACGTG  ACACGTTCGGAGAACA |
| shENO1#1 | T: GATCCGCGTGAACGAGAAGTCCTGCAACTCGAGTTGCAGGACT  TCTCGTTCACGTTTTTT |
|  | B: AATTAAAAAACGTGAACGAGAAGTCCTGCAACTCGAGTTGCAG  GACTTCTCGTTCACGCG |
| shENO1#2 | T: GATCCGCATTGGAGCAGAGGTTTACCCTCGAGGGTAAACCTCTG  CTCCAATGCTTTTTT |
|  | B: AATTAAAAAAGCATTGGAGCAGAGGTTTACCCTCGAGGGTAAAC  CTCTGCTCCAATGCG |
