# Supplementary material for: Targeting enolase 1 reverses bortezomib resistance in multiple myeloma through YWHAZ/Parkin axis
Source: J Biomed Sci. 2025 Jan 20;32:9. doi: 10.1186/s12929-024-01101-x (PMC11744840; doi:10.1186/s12929-024-01101-x)
Supplement: Supplementary file 5 — Supplementary Material 5. [file 12929_2024_1101_MOESM5_ESM.docx]

**Supplementary Materials and Methods**

**Cell viability assay**

Cell viability was determined by a Cell Counting Kit (CCK)-8 (Dojindo, Kumamoto, Japan). Cells were treated with indicated drugs following by adding 10 μL CCK solution at 37 °C for 2 h. Then the absorbance of each well was measured using a microplate reader. Analyzed the combined effect of drugs using the SynergyFinder 3 (<https://synergyfinder.fimm.fi>) [3]. The synergy score was used to evaluate the efficacy of the combination. Based on the user guide on the website, synergy score was assessed as follows: scores <−10: the interaction between two drugs was probably to be antagonistic; from −10 to 10: the interaction between two drugs was probably to be additive; and > 10: the interaction between two drugs was probably to be synergistic.

**Bioinformatics and statistics**

ENO1 protein expression data in MM were downloaded from the Gene Expression Omnibus (GEO) database. Experimental data were analyzed using the unpaired, two-tailed Student’s *t* test, Wilcoxon signed rank test, or Mann–Whitney *U*-test and the correlation was analyzed using a Spearman rank correlation test. Other statistical significance (*P* < 0.05) was assessed by the Student’s *t* test. Data were presented as the mean ± SD.

Gene Set Enrichment Analysis

The RNA-seq data of patients diagnosed with multiple myeloma (MM) were obtained from the GSE24080 dataset for subsequent gene set enrichment analysis (GSEA). Samples were stratified into high- or low-ENO1 expression groups according to the quartile distribution of ENO1 expression counts. Differential gene expression between two groups was assessed using R package “limma” [4], identifying genes with a *P*-value < 0.01 and a false discovery rate (FDR) < 0.05 as statistically significant. Then, these genes were further analyzed using the GESA function within the R package“clusterProfiler” [5], focusing on pathways from the Kyoto Encyclopedia of Genes and Genomes (KEGG) (http://kegg.jp). Finally, the normalized enrichment score (NES) was calculated to measure pathway enrichment. Visualization of the results was accomplished through ridge plots generated by the R package“clusterProfiler”. All analyses were conducted using R software （Version 4.2.1).

**RNA interference, plasmids and transfection**

The short hairpin RNA sequence of pGMLV vectors to deplete ENO1 are shown in Table S2. The pcDNA3-HA-ENO1 plasmid was constructed by Genomeditec Company (Shanghai, China). pCDH-Flag-YWHAZ were constructed by cloning the full-length YWHAZ cDNA into the pCDH vector. According to the manufacturer's instructions, Lipofectamine 3000 (Life Technologies, Carlsbad, CA, USA) was used to perform the transfections.

**Mitochondrial DNA gene expression analysis by qPCR**

Cells were re-suspended in 200 μL PBS containing Proteinase K, 0.2% SDS, EDTA (5mM) for 3h at 56 ℃. After isopropanol precipitation, 50 ng of DNA was amplified by qPCR using specific primers for 12S, D-LOOP and ACTB. Reactions were performed and analyzed as described above with a normalization to ACTB.

**5-ethynyl-2′-deoxyuridine (EdU) cell proliferation assay**

EdU cell proliferation assay was performed as described previously [6]. Briefly, MM cells (2 × 10 ^5^ cells/mL) stably transfected with shENO1 and shCtrl were collected, and EdU was incorporated using an EdU kit (Beyotime Biotechnology, Shanghai, China) according to the manufacturer's protocol.

**Transmission Electron Microscopy (TEM)**

After removing the culture medium, a 2.5% glutaraldehyde solution (P1126, Solarbio, China) was added to fix the cells for 2 h at RT. Subsequently, cells were collected and washed gently three times in PBS, and postfixed in 1% osmium tetroxide-PBS for an additional hour. Following dehydration through a series of graded ethanol solution, cells underwent critical point drying and were sputter-coated with 10% gold using a JEOL JFD-320 Cold Ice Dryer and a JFC-1600 Ion Sputterer. Observations were conducted using a transmission electron microscope (Hitachi H-7650) operating at an accelerating voltage of 80 kV.

**Immunoblotting**

Equal amounts of protein were separated on 8%, 10%, or 12.5% sodium dodecyl sulfate (SDS)–polyacrylamide gels and transferred to nitrocellulose membrane in tris-glycine transfer buffer (12 mM tris, 96 mM glycine, and 20% methanol). Membranes were blocked in 5% non-fat milk, incubated with primary antibodies overnight at 4°C, and then incubated with HRP-conjugated secondary antibodies for 1 h at room temperature. Images were captured with the Amersham Imager 600 (General Electric Company, USA) and densitometric analysis was performed using Image J software (http://rsbweb.nih.gov/ij/). Band intensities were normalized actin loading controls.

**Immunoprecipitation and co-immunoprecipitation assays**

For exogenous Co-IP, 293T cells were seeded into 10-cm tissue culture dishes at 10^6^ cells/dish. The next day, cells were transfected with 2 µg of either HA-tagged ENO1 or Flag-tagged YWHAZ plasmid DNA using Lipofectamine™ 3000 according to the manufacturer’s instructions. After 48 h of transfection, cells were collected and washed with ice-cold PBS, then lysed with ice-cold lysis buffer (20 mM Tris-HCl, pH 7.4, 150 mM NaCl, 1 mM EDTA, 1% Triton X-100, protease, and phosphatase inhibitor cocktails). Lysates incubated on ice for 30 min. The soluble fraction was isolated from the lysates by centrifugation at 12,000 rpm for 20 min at 4°C. 10% of the total lysate was used as input and the remaining lysate fractions were then incubated with 20 µl of Magnetic Agarose beads (MedChemExpress) overnight with rotation at 4°C. Immunoprecipitates were washed ten times with lysis buffer. Immunoprecipitated proteins were denatured by the addition of SDS-PAGE Protein Sample Loading Buffer (Beyotime) supplemented with and heating for 10 min at 100°C.

**Immunohistochemical (IHC) staining**

Briefly, tissue slides acquired from MM xenografts were deparaffinized and rehydrated, following by antigen retrieval, permeabilizated and 3% hydrogen peroxidase treatment. After being blocked with goat serum, slides were incubated with primary antibodies against Ki67 and ENO1 overnight at 4 °C. Then labeled by HRP (rabbit) second antibody at room temperature for 1 h. Finally, sections were developed in DAB solution under microscopic observation and counterstained with hematoxylin. Immunostained sections were scanned using Pannoramic SCAN Ⅱ.

**Tumor xenografts in nude mice**

Taking into account the effect size and standard deviation, the method for determining the appropriate sample size in animal studies is recommended by animal research committee. Prior to the experiment, male 4-week-old, BALB/c athymic nude mice were acclimated in the animal house for a week. Then, 3 × 10^6^ H929 cells resuspended in a total volume of 100 μL serum-free culture medium were implanted subcutaneously into each mouse. Once the tumors became measurable, BALB/c nude mice (5-week-old) were randomly assigned to 4 groups with 8 mice in each group and treated with bortezomib (1 mg/kg dissolved in 100 μL of the vehicle, thrice/week), AP-Ⅲ-a4 (10 mg/kg dissolved in 100 μL of the vehicle, twice/week), or a combination of both treatments. Tumor growth was measured by calliper measurements every 2 days and calculated using the formula 1/2 (length (mm)) × (width (mm))^2^. After the mice were euthanized, tumors were resected and formalin-fixed. Samples were paraffin embedded, cut at 4 μm and immunohistochemistry-stained for histological evaluation of target protein expression. All animal procedures were approved by the Institutional Review Board of the Shanghai East Hospital Ethics Committee (Approval No. TJBB00223101).

1. Hu, L., et al., *A novel M phase blocker, DCZ3301 enhances the sensitivity of bortezomib in resistant multiple myeloma through DNA damage and mitotic catastrophe.* Journal of Experimental & Clinical Cancer Research : CR, 2020. **39**(1): p. 105.

2. Xie, Y., et al., *Preclinical validation and phase I trial of 4-hydroxysalicylanilide, targeting ribonucleotide reductase mediated dNTP synthesis in multiple myeloma.* Journal of Biomedical Science, 2022. **29**(1): p. 32.

3. Ianevski, A., A.K. Giri, and T. Aittokallio, *SynergyFinder 3.0: an interactive analysis and consensus interpretation of multi-drug synergies across multiple samples.* Nucleic Acids Research, 2022. **50**(W1): p. W739-W743.

4. Ritchie, M.E., et al., *limma powers differential expression analyses for RNA-sequencing and microarray studies.* Nucleic Acids Research, 2015. **43**(7): p. e47.

5. Yu, G., et al., *clusterProfiler: an R package for comparing biological themes among gene clusters.* Omics : a Journal of Integrative Biology, 2012. **16**(5): p. 284-287.

6. Gao, X., et al., *A novel phosphoramide compound, DCZ0805, shows potent anti-myeloma activity via the NF-κB pathway.* Cancer Cell International, 2021. **21**(1): p. 285.
